# Components of aircraft life support systems interact with each other and the user

**DOI:** 10.3389/fphys.2022.969167

**Published:** 2022-09-26

**Authors:** F. Eric Robinson, Barbara E. Shykoff, Dan E. Warkander

**Affiliations:** ^1^ Naval Aerospace Medical Research Laboratory, Naval Medical Research Unit Dayton, Wright-Patterson Air Force Base, Dayton, OH, United States; ^2^ Oak Ridge Institute for Science and Engineering, Oak Ridge, TN, United States

**Keywords:** respiration, life support system (LSS), regulator, breathing resistance, work of breathing

## Abstract

The life support system in a tactical aircraft provides necessary supplemental oxygen to the aircrew. However, interactions among its various components may generate unexpected breathing loads. We focus here on the interactions between a regulator and breathing mask commonly used together in the U.S. Navy, the CRU-103 regulator and MBU 23/P mask, and some effects of the interactions on the user. The data reported were collected during a larger research effort examining potential physiological and cognitive effects of low regulator inlet pressures. Seventeen participants completed a series of tasks under mild exercise while breathing 40% O_2_ (balance N_2_) from an MBU-23/P mask supplied by a CRU-103 regulator with supply pressures 10, 6, 4, and 2 psig (CRU-103 specifications are for inlet pressures from 5 to 120 psig). Variables measured included flow to the mask and pressures at the regulator supply, in the hose to the mask, and in the mask. In addition to restricting inspiratory flow, low inlet pressure to the CRU-103 caused a counterintuitive overshoot in gas delivery pressure at end-inspiration, a mean increase of 1.5 cm H_2_O between the 10- and 2 psig conditions. The added pressure to the exhalation valve increased the expiratory threshold, the pressure to start expiratory flow, by approximately 2 cm H_2_O, increasing the effort needed to exhale.

## Introduction

Tactical aviation presents numerous physiological challenges for aircrew. First and foremost is exposure to altitude. In order to combat the potential effects of hypobaric hypoxia, aircrew breathe supplemental oxygen delivered through a mask supplied by a demand regulator, sometimes at a pressure slightly higher than that in the aircraft cockpit. The oxygen is often provided by an onboard oxygen generating system (OBOGS), a system of molecular sieve beds. The entire system of OBOGS, regulator, mask, and associated piping and connectors is called the life support system (LSS). The aircraft LSS is designed to meet performance standards, e.g., MIL STD 3050 (Department of Defense, 2015). However, it inevitably increases breathing resistance and other breathing loads relative to maskless breathing. Aircrew may compensate for, or have reflex responses to, respiratory loads; testing is necessary to identify any such responses in order to mitigate unanticipated physiological effects and maximize aircrew health and performance. This study was conducted as part of a broader effort to examine the potential physiologic effects of breathing loads imposed by some aspects of one LSS.

Though this particular study focuses on a combination of mask and regulator commonly used in tactical aviation in the U.S. Navy, we first describe a generic aircraft LSS supplied by an OBOGS. The molecular sieve beds of the OBOGS bind nitrogen reversibly when they are at high pressure and release it when they cycle to low pressure, and every OBOGS has at least two beds. After it is cooled, high-pressure engine bleed air is fed to a bed and oxygen-enriched gas (up to roughly 94% O_2_, 6% argon) flows out the other side. The concentrated oxygen, at a pressure only slightly lower than that at the OBOGS inlet, passes downstream through the plumbing of the LSS to the regulator which supplies the breathing mask. Concurrently, the other bed or beds are purged with a small flow of oxygen while they are exposed to the low ambient pressure outside the aircraft to unload the bound nitrogen. Periodically the beds switch, with a newly purged bed beginning to receive pressurized engine bleed air while the expended bed starts a purge cycle for regeneration.

The pressure supplied to the pilot’s breathing regulator depends on the pressure of the engine bleed air supplying the OBOGS. The OBOGS outlet pressure will be low if the engine bleed air pressure is low (low engine speed). If the OBOGS outlet pressure is low, large inspiratory flow needs of the pilot may propagate upstream to the OBOGS. The oxygen content of the gas depends on nitrogen loading and pressure in the active OBOGS sieve bed. Regeneration rate increases with increasing aircraft altitude. Thus, OBOGS output oxygen content may be lower than maximum at low altitude if the purging cycle cannot completely regenerate the bed. It also may dip if the pilot respiratory demand pulls gas too rapidly from the OBOGS or otherwise decreases pressure within the OBOGS. This study deals only with gas pressures and flows, not with oxygen concentrations.

The breathing regulator used varies across the branch of service and type of aircraft. In the U.S. Navy, tactical aircrew in the T-45 and F-18 aircraft use a CRU-103 regulator (Eaton, Orchard Park, NY) mounted on a chest harness. There are currently two masks used by U.S. forces in tactical aircraft, the MBU-20/P and MBU-23/P masks (Gentex, Zeeland, MI), both of which use the same valves and facepiece. The U.S. Navy uses the MBU-23/P.

The regulator, mask valves, and pilot respiratory characteristics interact to determine mask pressures and gas flow to the pilot, as follows. The CRU-103 supplies the pilot’s mask with gas on demand. It responds mechanically, releasing gas when the regulator output pressure decreases below its set-point pressure and stopping flow when the pressure at the regulator output port reaches or exceeds that pressure. When the pilot starts to inhale, pressure in the mask decreases. When the pressure in the mask is lower than the pressure in the regulator output hose, the mask inhalation valve opens. The flow of gas into the mask from the hose decreases the regulator output pressure and initiates flow from the regulator. When the pilot reaches the end of the inhalation, the pressure in the mask rises. The inhalation valve closes when the pressure matches or just exceeds that in the regulator output hose. The pressure at the regulator outlet returns to its set-point, and regulator outlet flow ceases. The increased mask pressure also opens the exhalation valve. The pressure to open the exhalation valve is slightly higher than that in the inhalation hose; a compensation tube in the mask applies the hose pressure present just outside the inhalation valve to the back of the exhalation valve. The compensation tube pressure acts in addition to that of the spring which pushes the valve closed. The functions of the regulator and valves are not instantaneous - gas must move through the system and mechanical parts and gas must be accelerated from a stand-still and deaccelerated to a stop. The mechanical requirements of moving gas through the regulator and mask impose additional breathing restrictions and alter timing relative to breathing ambient air without a mask. Additionally, the tubing and its bends and fittings provides flow resistance (pressure drop as a function of flow) when gas is moving.

Disruptions to gas supply can impose additional respiratory loads. Although the specifications for the CRU-103 indicate operating regulator inlet pressures from 5 to 120 psig (Specification sheet 100-001-702, CRU-103 Oxygen Regulator.pdf (menlosecurity.com)), its performance is known to be suboptimal at the lower range of those input pressures (Gordge, 2004). As is alluded to above, during conditions of low engine output, engine bleed air may not be delivered to the OBOGS at sufficient pressure to maintain proper operation of the CRU-103. When the CRU-103 supply pressure is low, the regulator operation depends in a complex way on flow demand. A reduction in the supply pressure to the CRU-103 does not cut off gas flow to the pilot entirely. Low flows may be freely available, but higher flows may be available, if at all, only with extreme mask pressures. For CRU-103 inlet pressure that is maintained at 2 psig, flow is limited to 1 L/s, while with 6 psig, the maximum available flow is 2.2 L/s (Gordge, 2004), nominally more than adequate for normal breathing but not for high demand. However, when the regulator inlet pressure is marginal, breathing draws the pressure down. Additionally, even at flows low enough that they are not limited because of low supply pressure, resistance to flow is higher when regulator supply pressure is lower. Whether the gas flow at a given CRU-103 inlet pressure is adequate depends on the breathing demands of the person using the regulator; breathing demands vary across different phases of flight (Gordge, 1993) and among pilots.

During ground operations of F/A-18 and F-14 aircraft in which flow was measured for a total of 8 pilots, about 45% of breaths had peak flows greater than or equal to 1 L/s but only 1% of breaths reached 2 L/s. For routine flight, peak flows in 42 aircrew were at or above 1 L/s for approximately 75% of breaths, but at or above 1.4 L/s for only 10% of breaths, with a mean peak flow of approximately 1 L/s (59 ± 17 L/min; Gordge, 1993). Pilots typically have a higher mean peak flow of 78 ± 25 L/min during routine flight operations than during ground operations (Gordge, 1993).

Other conditions like compromised mask valve function also may lead to either reduced availability of gas to the pilot or brief but noticeable impediments to breathing. Systems that cannot keep up with these demands will fail to provide adequate breathing gas to the aircrew, leading to respiratory disruptions and potential changes in CO_2_ levels or other physiological effects.

The nature of the physiological response to a respiratory load depends on the timing, duration and type of impediment to normal breathing. Low-level resistive loads are generally well-tolerated, with no change in breathing except for the necessary increase in pressures, or small changes in breathing pattern with no appreciable decrease in CO_2_ control (Bentley et al., 1973; Deno et al., 1981; Caretti and Whitley, 1998; Shykoff and Warkander, 2011; Shykoff and Warkander, 2012). Higher resistive loading or moderate resistive loading at high physical work rate will cause minute ventilation to decrease and metabolically-produced CO_2_ to accumulate in the body (Cerretelli et al., 1969; Shykoff and Warkander, 2011; Shykoff and Warkander, 2012). However, the effects of impediments caused by constant low supply pressure to the CRU-103 have not been defined, and physiological adjustments to sudden, unexpected changes in gas delivery are not fully explored. Inspiratory obstructions can affect muscular output (Eckert et al., 2008) and duration of inspiratory effort (Romaniuk et al., 1993). This potentially reduces the total volume of the subsequent breath (Zin et al., 1983). Inspiratory threshold loading, that is, the need to decrease inspiratory pressure considerably before flow starts, causes its own set of reflex responses and a sensation of difficulty inhaling (Chen and Yan, 1999; Yan and Bates, 1999).

MIL STD 3050 (Department of Defense, 2015) specifies the set of maximum allowable mask pressures for a set of peak flows, producing the so-called “trumpet curve” of mask pressure as a function of peak flow. ISO 16976-4 (2019) describes maximum acceptable work of breathing for respiratory equipment in non-aviation environments. This study examined the effect of baseline and reduced CRU-103 supply pressures on system behavior when operating at the lower bounds of MIL STD 3050, as well as individual differences in the respiratory behaviors of users in response to changes in the breathing system. We first used a breathing simulator to quantify CRU-103 performance relative to MIL STD 3050 at multiple supply pressures. Next, we evaluated human respiratory and physiological responses to breathing using a CRU-103 supplied at multiple different pressures. The regulator, mask, and human interact with one another such that breathing patterns both respond to and influence changes in the operation of the regulator and mask.

## Materials and methods

### Unmanned testing

We first characterized the purely resistive behavior of the CRU-103 at various supply pressures in order to identify test pressures for human data collection. We utilized a piston-operated breathing simulator (Series 1120 flow/volume simulator; Hans Rudolph, Shawnee, KS) set to breathe with sinusoidal flow patterns through a headform (Mine Survival Inc., Panama City Beach, FL). A standard flight helmet (HGU-55/P; Gentex, Zeeland, MI) and breathing mask (MBU 20/P; Gentex) were fitted to the headform, with the mask connected to a CRU-103 regulator by a standard aviator’s breathing hose. The mask was tapped for measurement of pressure. The regulator was certified by qualified technicians and replaced according to the recommended 90 days inspection cycle. Compressed gas was delivered at pressure to an 8 L plenum (accumulator) and then to the regulator inlet.

We recorded pressures at the supply to the CRU-103 (plenum output), at the output from the CRU-103, and from the mask. We measured flow between the breathing simulator and the headform neck using a pneumotachometer (Series 4830, flow range 0 to ±400 L/min, Hans Rudolph). All pressures, including those generated by the flow, were measured using pressure-compensated, amplified, ratiometric pressure transducers (SSC series, Honeywell SIT, Fort Mill, SC). All signals were sampled at 100 Hz using software written in-house (LabView, National Instruments, Austin, TX).

**FIGURE 1:**
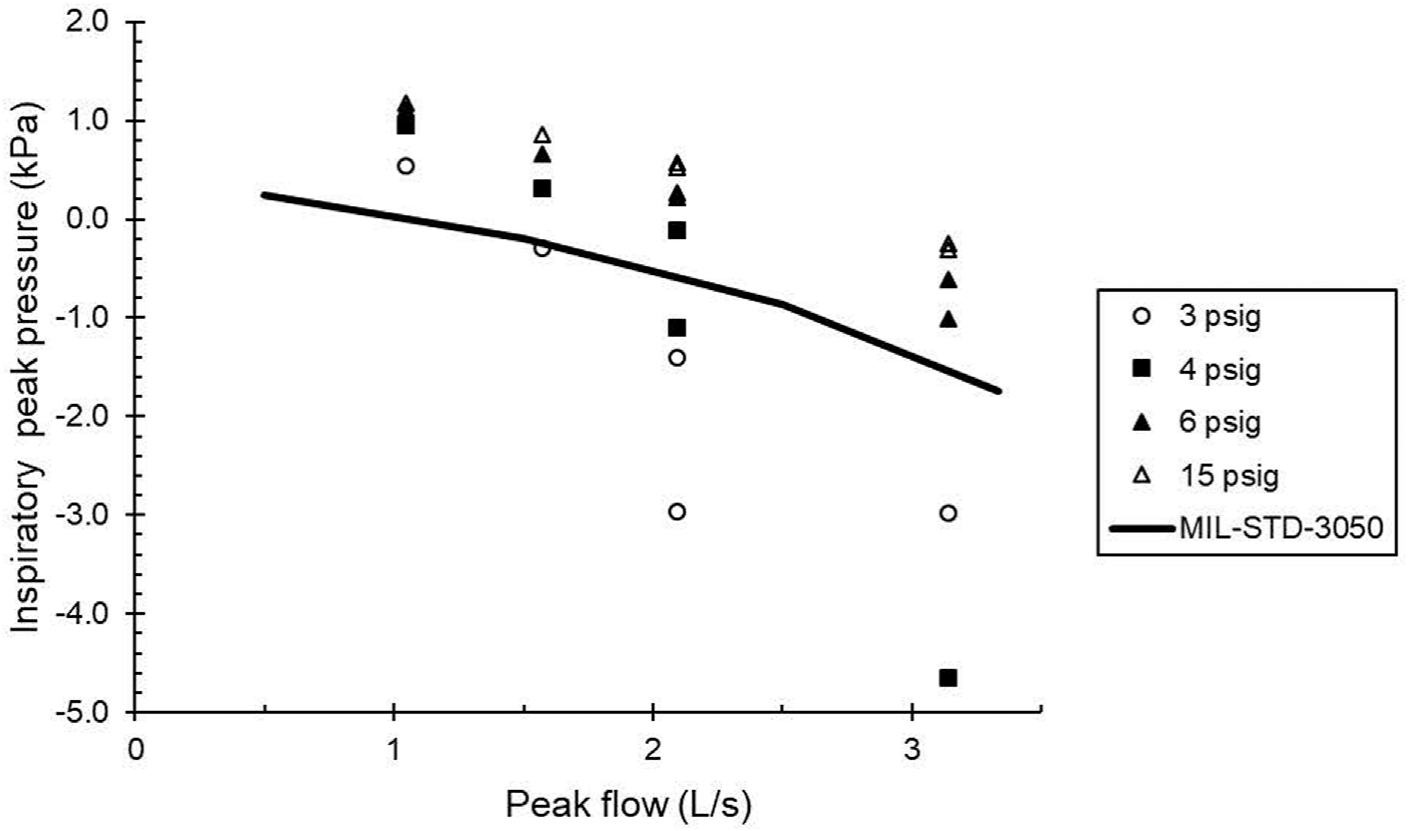
Comparison of minimum inspiratory pressures (peak inspiratory pressure) as a function of flow to the inspiratory portion of the “trumpet curve” of MIL STD 3050. Solid line: standard. Symbols: measured values. Data are labeled with their nominal supply pressures. Points above the solid line are within the standard. Peak flows with two separate pressures plotted were generated with different tidal volume—frequency combinations set on the breathing simulator.

Testing using the breathing simulator indicated that the CRU-103 met MIL STD 3050 if the supply pressure was 6 psig or greater (Figure 1). The CRU-103 met the ISO 16976-4 work of breathing standard, that inspiratory work of breathing per tidal volume (WOB_i_/V_T_) should not exceed 0.9 kPa, for minute ventilations up to 60 L/min with sinusoidal breathing and an inlet pressure of 6 psig.

The unmanned data indicate a serious risk of flow limitation during regular operations if the CRU-103 inlet pressure is 2 or 4 psig. (Both of these are below the nominal operating range, but may occur in an aircraft during engine idle, for example). For those regulator supply pressures, peak attainable flows are approximately 1 and 1.4 L/s, respectively. Even for a regulator inlet pressure of 6 psig (above the nominal minimum supply pressure of 5 psig), the maximum flow delivered by the CRU-103 is 2 L/s (120 L/min), and flow limitation during normal breathing is still probable. Based on Gordge (2004), there is no significant risk of flow limitation during normal breathing if the CRU-103 inlet pressure is 10 psig, but a possible limitation for very sharp inhalations remains.

With 6 psig or more at the CRU-103 regulator inlet, peak pressures and flows satisfied MIL STD 3050. However, with a regulator inlet pressure of 4 psig, pressure flow characteristics were marginal for MIL STD 3050. Based on these results, we selected 10 psig as our baseline supply pressure to the CRU-103, with test pressures of 6, 4, and 2 psig to represent values expected to be acceptable, marginal, or inadequate for meeting anticipated breathing demand.

### Human testing

Phase 2 utilized a 3 × 3 [(CRU-103 inlet pressure) x (test phase—baseline, test pressure, recovery)] single-blind, repeated measures design. This study was approved by the Naval Medical Research Unit Dayton (NAMRU-D) Institutional Review Board.

#### Participants

We solicited volunteers from the active duty population stationed at Wright Patterson Air Force Base, OH. Thirteen men and four women, ages 19 to 50 (median 35, 1st to 3rd quartiles 27–37) volunteered. Participants were ineligible if they reported a history of medical conditions or lifestyle habits that may have affected safety or study results (e.g., heart/circulatory problems, recent pneumonia, asthma, sickle cell trait, neurological conditions, tobacco use).

#### Instrumentation and data collection

Compressed gas was delivered at a pressure of 10, 6, 4, or 2 psi to an 8 L plenum, and then to a CRU-103 regulator *via* a ½ inch pipe. The breathing gas was 40% O_2_, balance N_2_, which approximates the oxygen concentration delivered on the ground by one OBOGS-equipped aircraft (T. Castro, personal communication, 6 April 2018). System pressures were measured as during unmanned testing. As with unmanned testing, the CRU-103 was certified by qualified technicians and replaced according to the recommended 90 days inspection cycle. From the CRU-103, the breathing gas passed through a pneumotachometer (Series 4830, flow range 0 to ±400 L/min, Hans Rudolph) then through a standard aviation breathing hose to a Gentex MBU 23/P flight mask, the mask model most commonly used in the U.S. Navy. All mask valves were in new condition at the beginning of the study. As in unmanned testing, ports added to the mask allowed measurement of mask pressure and gas composition.

In addition to pressure and flow, we captured physiological, cognitive performance, and subjective data. These results are not reported in this paper, but we describe the equipment and measures briefly here for completeness and to help clarify our description of the procedure below. We captured CO_2_ in the mask utilizing a fast-response NDIR analyzer (GA-22, iWorx, Dover, NH) and transcutaneous levels of oxygenation and CO_2_ using a TCM4/COMBI m84 monitor (Radiometer, Copenhagen). Heart rate (HR) and respiratory rate were measured using a Zephyr puck and harness (Zephyr, Medtronic, Minneapolis, MN), with data collected on custom software (COG Pack; originally developed by the Air Force Research Laboratory for Menke et al., 2015). Study participants performed a tracking task to assess aspects of cognitive performance related to sustained attention and psychomotor control. They used a joystick (HOTAS Cougar, Thrustmaster, Carentoir, FR) to keep a randomly moving cursor as close as possible to a target in the center of a computer screen (LCD monitor, Dell, Round Rock, TX). In addition to the tracking task, participants reported subjective assessments of their experience breathing through the regulator and mask. Study participants were instructed to press a green button located next to the joystick at any time if they noticed any change in the apparent pressure of the breathing gas, a yellow button if they noticed any physiological symptoms they thought were related to the exposure, and a red button if the symptoms reached a point where they did not wish to continue (i.e., they would likely declare an in-flight emergency, or pull their car to the side of the road for those who were not flight crew). Study participants also periodically were asked for a verbal subjective assessment of their overall workload using Borg’s Rating of Perceived Exertion (RPE) scale (Borg, 1970). This scale is designed to correlate with estimated HR and ranges from 6 (no exertion) to 20 (maximal exertion).

Respiratory demand was increased by exercise at 1 kp and 50 rpm (50 W) on a cycle ergometer (Ergomedic 828E, Monark, Vansbro, Sweden), generating a minute ventilation of approximately 25 L/min.

#### Procedure

Each study participant reported to NAMRU-D on three separate occasions, with testing sessions separated by at least 24 h. A different test condition was administered at each visit, with the order of test conditions counterbalanced across participants. The same procedure was followed each time, with the exception of obtaining informed consent at the beginning of the first visit. Female participants were given a urine test each visit to rule out pregnancy. Participants were fitted with the transcutaneous CO_2_ sensor and the Zephyr harness. The cognitive task was explained and participants were required to practice for 150 s prior to each exposure. They were also introduced to or reminded of the subjective response buttons and the Borg RPE scale. Study participants then donned the flight helmet and mask and began the test session.

**FIGURE 2:**
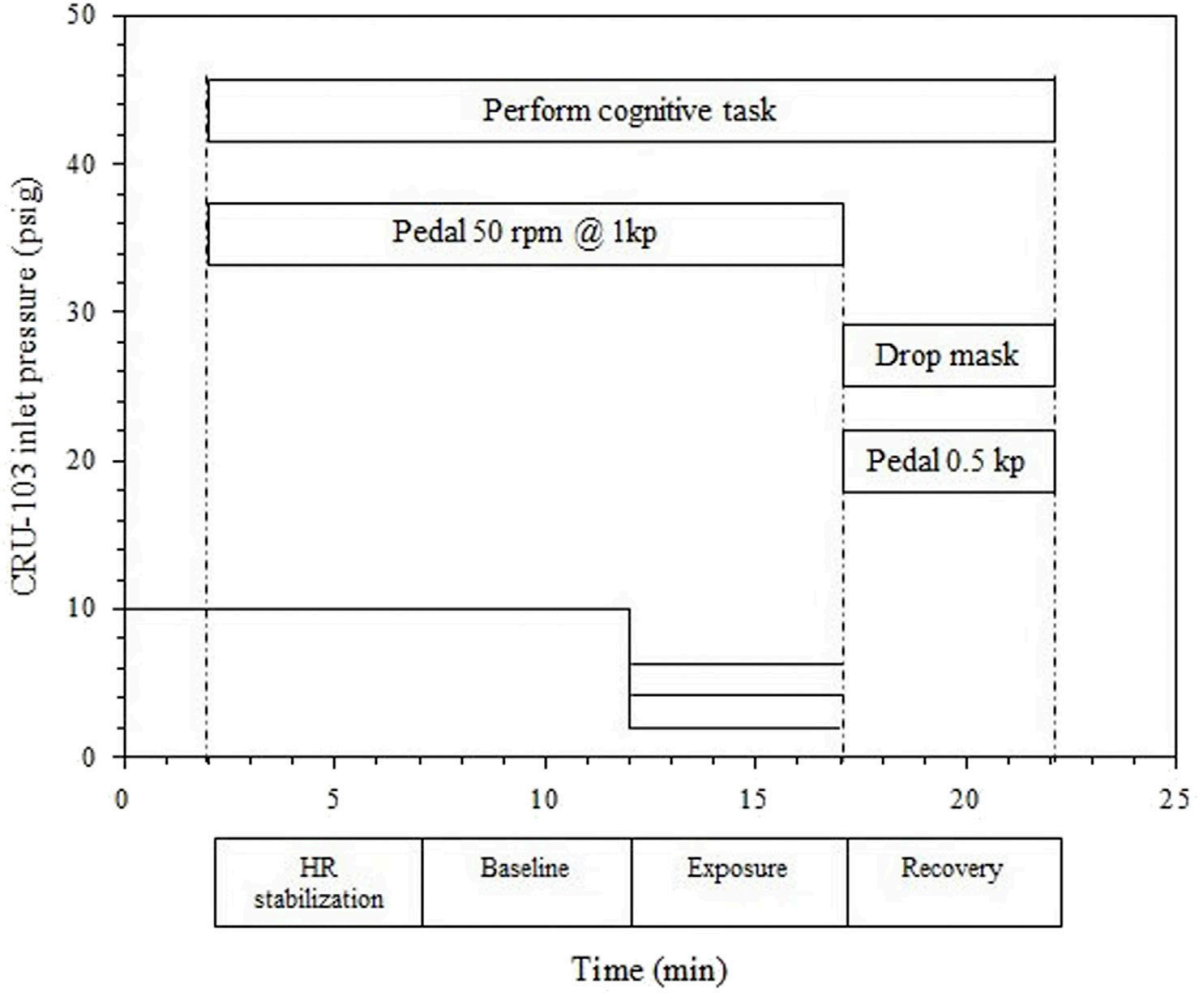
Exposure profiles for Phase 2 testing in the 6, 4, and 2 psig conditions. Each horizontal line between minutes 12 and 17 represents a separate exposure level, with a single level delivered per study session.

Each test profile consisted of a two minute cycling warm up against a force of 0.5 kp with pedal cadence uncontrolled, 15 min at an ergometer setting of 1 kp with pedal cadence 50 RPM for a work rate of 50 W, and a 5 min recovery period with the ergometer at 0.5 kp and pedaling cadence not controlled. For the period at 50 W, a metronome was used to help participants maintain cadence. The 15 min at 50 W consisted of a 5 min HR stabilization period, a 5 min baseline period, and a 5 min exposure period with one of the lower gas supply pressures (6, 4, or 2 psig). The participant breathed room air during recovery (Figure 2). The CRU-103 inlet was supplied with 10 psig during the warm-up, HR stabilization, and baseline periods. Its inlet pressure was manually controlled throughout to maintain the average pressure as close to the target as possible despite variability in participants’ breathing patterns (e.g., sudden large inhalations). The cognitive tracking task continued from the start of the stabilization period until the end of the recovery. Participants provided a single rating on the Borg RPE scale at minutes 1, 3, and 5 within each of the baseline, exposure, and recovery phases (nine total ratings throughout the profile).

A participant dropped the mask and began the recovery period when the time limit was reached or to terminate early. Criteria for early termination were pushing the red button or otherwise indicating a wish to stop the exposure, or reaching 85% of age-predicted maximum HR. (One person terminated early by pushing the red button. No one reached a heart rate limit.)

After the recovery periods, participants completed debriefing questionnaires on which they reported the nature of any physiological symptoms. We asked participants to list any symptoms in order as best as they could recall.

### Analysis

We conducted multiple analyses to investigate potential effects of reduced regulator inlet pressure. We first assessed the effects of supply pressure on the operation of the regulator and/or mask, and then identified correlations with user respiratory patterns. Mask pressure was used to identify inhalation/exhalation. Inlet flow was used to calculate tidal volume, respiratory timing, breathing frequency, and minute ventilation.

Statistical analyses of the respiratory averages for each participant under each condition were performed using SPSS version 16. For each variable considered, one minute averages for the minute that ended 30 s before the end of the test phase (baseline, exposure, and recovery) were compared across the four regulator inlet pressures. Differences were considered to be significant with *α* = 0.05.

The validity of assuming that the data were normally distributed was assessed using Q-Q plots and histograms. Data that were normally distributed were analyzed using repeated measures ANOVA with simple contrasts comparing the test conditions to the baseline condition. Corrections for violations of sphericity were applied as appropriate. Data with distributions very different from normal, for example, multi-modal distributions, were examined using the non-parametric pairwise Wilcoxon signed rank test, with Bonferonni correction for multiple comparisons when necessary.

When the standard deviations of normally-distributed data appeared to be greater for one condition than for another, variances were compared using an F-test. For similar data tested non-parametrically, large interquartile differences were described qualitatively without statistical testing.

Some pairs of variables, for example, mask pressure and inspiratory flow, were checked for relationships. Pearson correlation coefficients for pairs of variables were compared to the critical correlation coefficient for *α* = 0.05 and the number of samples, the value of which is 0.482 for *n* = 17 and 0.497 for *n* = 16 (the 4 psig condition). No adjustments were made for non-normal probability distributions or for small sample size.

## Results

### Flow limitation at low CRU-103 supply pressures

The CRU-103 was able to meet MIL STD 3050 for all participants when the regulator input pressure was 10 psig. However, performance decreased as regulator supply pressure decreased, such that the standard was not met for 2 participants at 6 psig, was not met for 10 participants at 4 psig, and was not met for any participants at 2 psig. Flow limitations likely account for the difficulty in meeting MIL STD 3050 at lower supply pressures. Although the respiratory demands were only moderate, across 3 min of breathing, with regulator inlet pressure 2 psig, 16 participants reached the flow limitations of the CRU-103 in many to most breaths and one in only one breath; with regulator inlet pressure 4 psig, five participants hit flow limitations in many breaths and five in 1–5 breaths; with 6 psig at the regulator inlet, two participants frequently reached flow limitation, three “touched” flow limitation frequently but didn’t increase inspiratory pressure very far into the limited region, and three participants reached flow limits in 1–3 breaths; and with 10 psig at the inlet, two participants reached flow limitation for a single breath.

**FIGURE 3:**
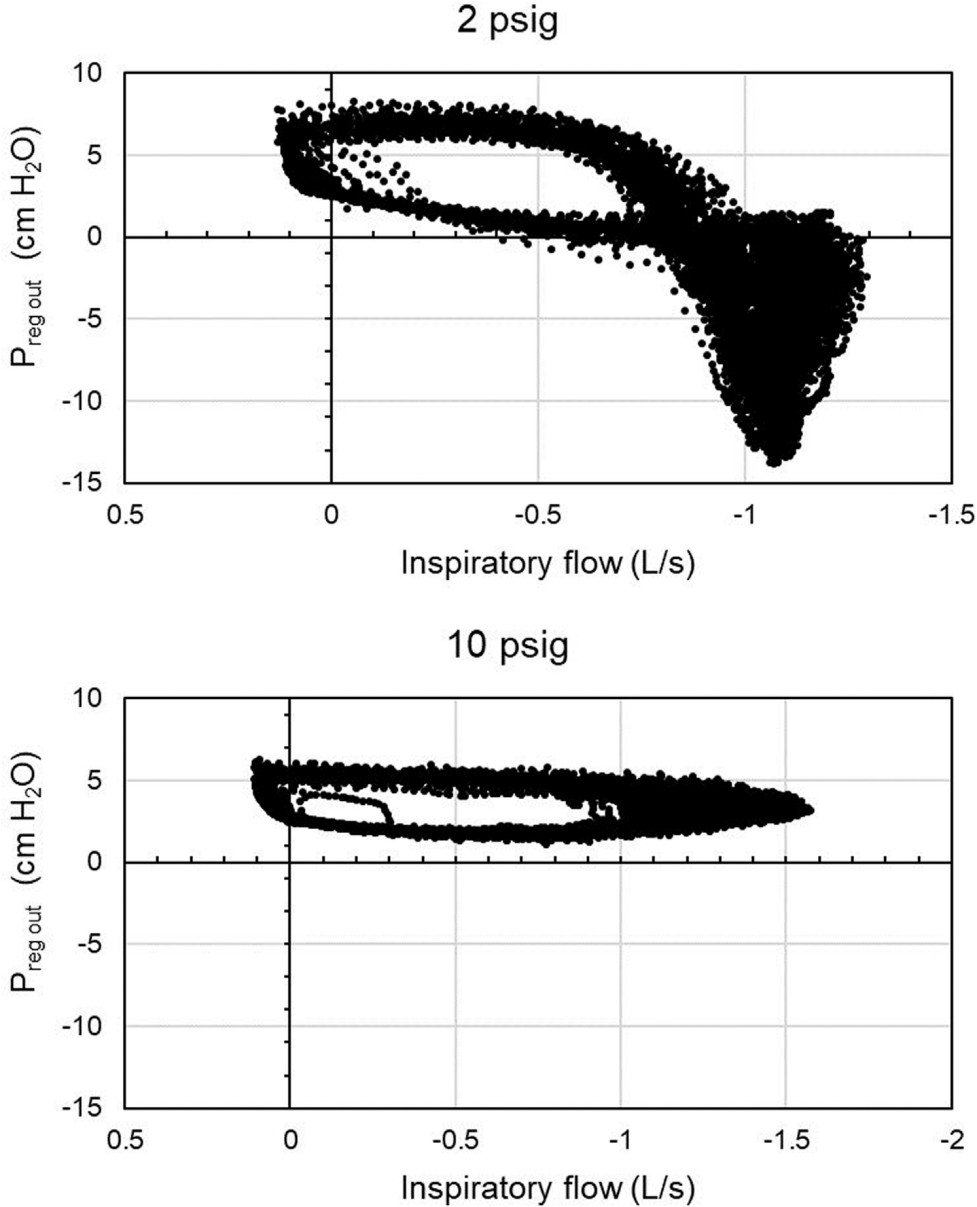
Regulator outlet pressure relative to room pressure showing flow limitation from the regulator. Data are from participant 3 with a nominal regulator inlet pressure of 10 and 2 psig. Regulator inlet pressure in reality varied from breath to breath depending on breathing characteristics. Inspiratory flow is given in negative values to correspond to negative mask pressures, but is plotted here with magnitude increasing to the right. At 2 psig supply pressure, the CRU-103 could not support flows greater than roughly 1.25 L/s, resulting in negative pressures at the regulator outlet relative to the room as the participant drew air out of the breathing hose faster than the regulator could deliver it.

An example is shown in Figure 3, where all values of regulator outlet pressure are plotted against their simultaneously-occurring inspiratory flow. Response curves differ among breaths because the supply pressure also varied somewhat. The minimum regulator outlet pressures always occurred during inspiration. Wilcoxon’s sign test showed that the median minimum output pressure decreased across inlet pressures (Table 1; medians and values for 3rd-1st quartiles for each condition in cm H_2_O are listed alongside the test comparison). The mean inspiratory flow into the mask had a lower median magnitude for the 2 psig regulator inlet pressure condition than for the 10 psig condition (*Z*(15) = 2.769, *p* < 0.02). Likewise, pairwise Wilcoxon signed rank tests (Table 2; medians and values for 3rd-1st quartiles for each condition in cm H_2_O are listed alongside the test comparison) showed that all other regulator inlet conditions had lower median minimum mask pressures than did the 10 psig condition.

**TABLE 1:** Wilcoxon sign test of minimum regulator outlet pressures for different regulator inlet pressures.

Comparison	Standardized test statistic *Z*	*df*	*p*-value, Bonferroni corrected
10 psig (2.04; 0.32) to 6 psig (1.69; 0.34)	3.48	15	<0.001
10 psig (2.04; 0.32) to 4 psig (1.15; 1.37)	3.52	15	<0.001
10 psig (2.04; 0.32) to 2 psig (-4.49; 8.80)	3.62	15	<0.001

**TABLE 2:** Wilcoxon sign test of minimum mask pressure for different regulator inlet pressures.

Comparison	Standardized test statistic *Z*	*df*	*p*-value, Bonferroni corrected
10 psig (0.02; 0.55) to 6 psig (-0.37; 0.57)	2.96	16	<0.001
6 psig (-0.37; 0.57) to 4 psig (-1.15; 1.70)	3.26	15	<0.001
6 psig (-0.37; 0.57) to 2 psig (-5.78; 10.58)	3.53	15	<0.001

### Mask and regulator interactions

Our finding that regulator output flow during inhalation was reduced at lower supply pressures is not surprising. However, we also found unexpected effects during expiration. The maximum regulator outlet pressure occurred early in expiration, just after the inspiratory valve closed. Analysis of variance after correction for lack of sphericity showed a significant effect of regulator inlet pressure on maximum regulator outlet pressure; *F*(2.28) = 24.66, *p* < 0.001. A simple contrast indicated that all lower regulator inlet pressures differed from 10 psig (Table 3; means/SD for each condition in cm H_2_O are listed alongside the test comparison). The maxima increased as the regulator inlet pressures decreased, as did their variability. The values from 4 psig and 2 psig were significantly more variable than those for 10 psig (*F*-test, Bonferroni–corrected *p* [*p*_*B*_] < 0.01), and those from 2 psig were significantly more variable than those for 6 psig (*F*-test: *p*_*B*_ < 0.01).

**TABLE 3:** Contrast statistics from repeated measures ANOVA analysis of maximum regulator outlet pressures.

Comparison	*F* Statistic for significant differences	*df*	*p*-value
10 psig (5.21; 0.26) to 6 psig (5.48; 0.58)	9.26	15	<0.03
10 psig (5.21; 0.26) to 4 psig (5.92; 0.85)	17.5	15	<0.01
10 psig (5.21; 0.26) to 2 psig (6.85; 1.02)	43.7	15	<0.001

**FIGURE 4:**
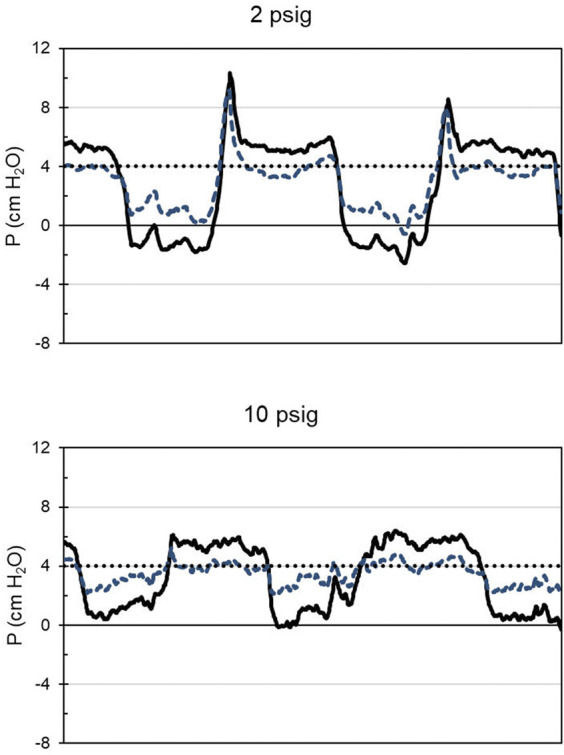
A sample of mask and regulator output pressures relative to room pressure as a function of time for 10 s. Participant 16, with the CRU-103 supplied with 2 or 10 psig. Solid black line: mask pressure. Dashed blue line: regulator outlet pressure. Dotted black line: nominal safety pressure. Mask pressures below safety pressure represent inspiration, and those above, expiration. Note pressure spikes at the start of expiration when the regulator inlet pressure is 2 psig.

**FIGURE 5:**
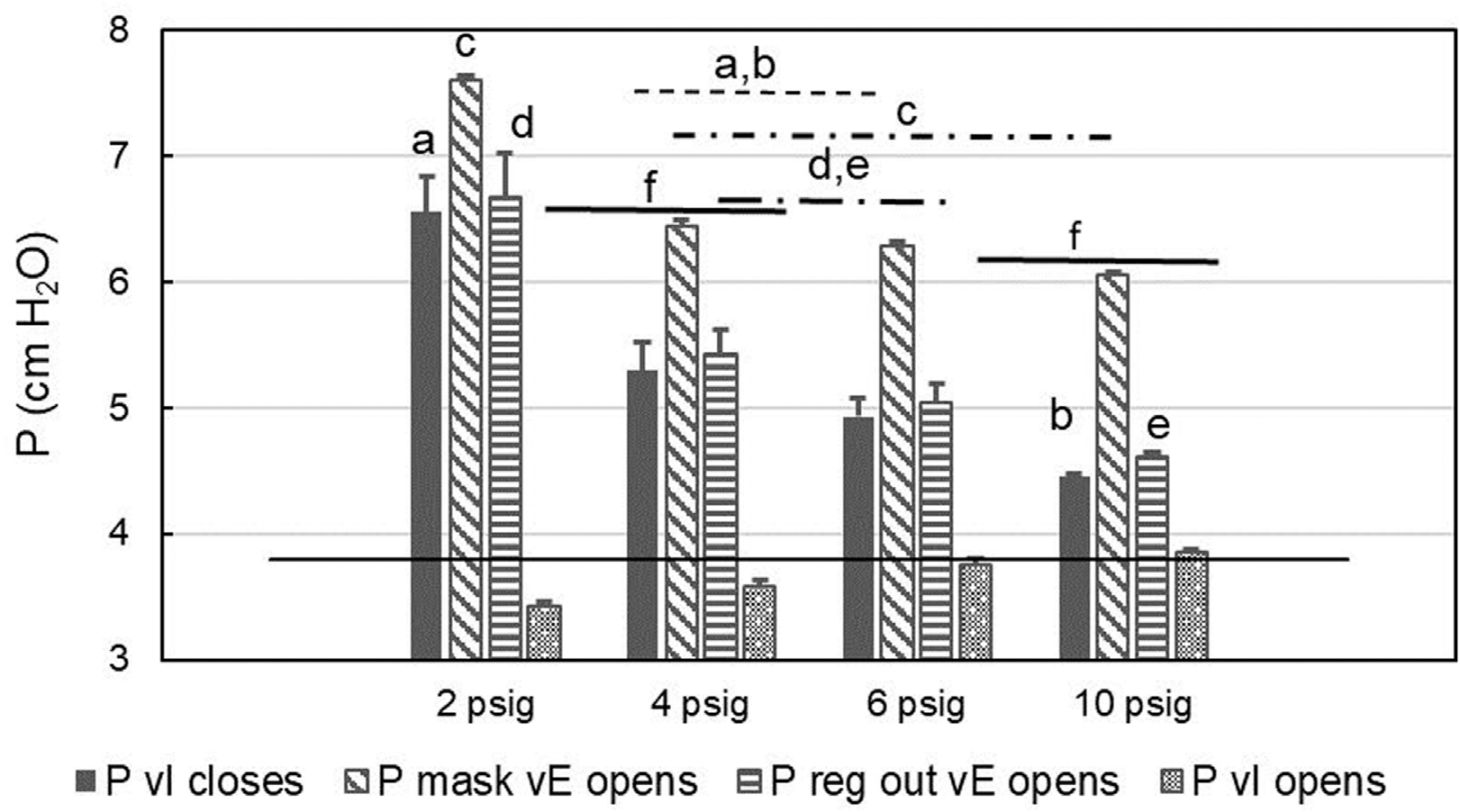
Mask and regulator output pressures when valves open or close. Means and SE across participants (200 s averages of breath-by-breath values) are shown. P = pressure, vI = inspiratory valve, vE = expiratory; black line: nominal safety pressure. Letters and horizontal lines above the bars indicate significant differences. The letters identify individual columns/comparisons and the horizontal lines indicate averaged values across conditions. For example, Column “a” (P vI closes at 2 psig) is significantly different from the mean of the same variable at 4 and 6 psig (indicated by the dashed line with “a,b” above it), which in turn is significantly different from Column “b” at 10 psig. Similarly, the mean of “P vI opens” across 2 and 4 psig is different from the mean across 6 and 10 psig.

Counterintuitively, it appears that at low supply pressures (below the minimum specified pressure) the CRU-103 “overshoots” and delivers a burst of elevated pressure as inspiratory flow stops before the start of exhalation. The elevated pressure appears to propagate through the mask compensation tube to influence the operation of the exhalation valve. Spikes in mask pressure in early expiration (Figure 4) indicate that increased pressure was necessary to open the mask exhalation valve. The opening and closing pressures for the valves in the mask across CRU-103 supply pressures can be seen in Figure 5. For a detailed explanation of mask exhalation valve function under varied conditions, including recordings of the pressure inside the expiratory valve, see Warkander and Arnold, (2020).

**FIGURE 6:**
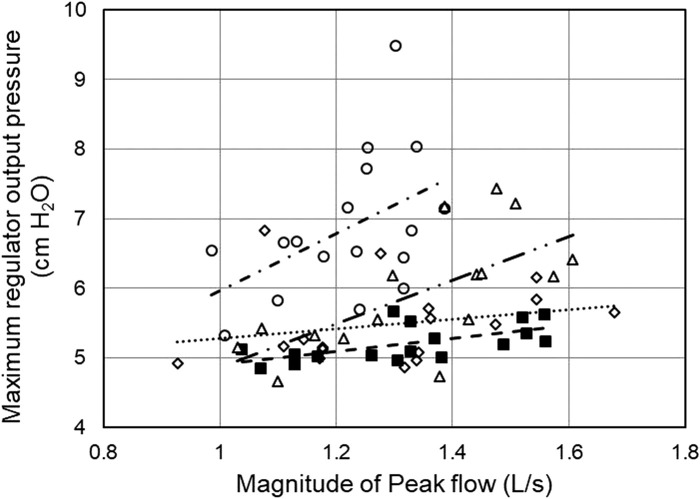
Correlation of maximum regulator outlet pressure with peak inspiratory flow. Data are one-minute averages from individual participants. Best fit lines are shown. Conditions: 10 psig ■ and - -; 6 psig ◇ and···; 4 psig, Δ and—· –; and 2 psig ○ and –· ·–. Note that all values shown on the *y*-axis represent regulator overshoot; the axis starts at 4 cm H_2_O.

Regulator overshoot appears related to the respiratory activity of the user. The maximum regulator output pressure increased with the magnitude of the peak inspiratory flow (Figure 6). The correlation coefficients *r* were = 0.61 (*t* = 2.95, *df* = 15, *p* = 0.01), 0.24 (*t* = 0.94, *df* = 15, *p* > 0.3), 0.66 (*t* = 3.33, *df* = 14, *p* < 0.01), and 0.47 (*t* = 2.06, *df* = 15, *p* < 0.06), for regulator inlet pressures of 10, 6, 4, and 2 psig respectively, with significant correlations for 10, 6, and 4 psig at the regulator inlet. Similar correlations existed between maximum regulator outlet pressure and minute ventilation, (*r* = 0.46, *t* = 2.02, *df* = 15, *p* = 0.061); (*r* = 0.60, *t* = 2.85, *df* = 15, *p* < 0.02); (*r* = 0.58, *t* = 2.68, *df* = 1, *p* < 0.02); and (*r* = 0.53, *t* = 2.44, *df* = 15, *p* < 0.03) for regulator inlet pressures of 10, 6, 4, and 2 psig, respectively. Note that this correlation was statistically significant for 2, 4, and 6 psig. In general, regulator overshoot during expiration increased with increasing magnitude of peak flow or minute ventilation during inspiration, and also with decreasing regulator supply pressure below 6 psig.

### Respiratory changes across supply pressures

Median WOB_i_/V_T_ increased progressively with decreased regulator inlet pressure (Table 4; medians and values for 3rd-1st quartiles for each condition in kPa are listed alongside the test comparison). We did not identify any significant changes in minute ventilation, tidal volume, or total breath duration across the CRU-103 supply pressures used in this study. However, average inspiratory duty cycle (T_i_/T_tot_; the fraction of a breath used for inhalation), showed a significant effect of regulator inlet pressure (after correction for non-sphericity, *F*(1,1.99) = 14.36, *p* < 0.001). Inspiratory duty cycle was significantly longer for the 2- than for the 10 psig regulator inlet pressure condition (*t* = 4.58, *p* < 0.001).

**TABLE 4:** Wilcoxon sign test of WOB_i_/V_T_ for different regulator inlet pressures.

Comparison	Standardized test statistic *Z*	*df*	*p*-value, Bonferroni corrected
10 psig (0.35; 0.07) to 6 psig (0.41; 0.13)	3.62	16	<0.001
6 psig (0.41; 0.13) to 4 psig (0.53; 0.29)	2.53	15	<0.01
6 psig (0.41; 0.13) to 2 psig (0.98; 0.63)	3.62	16	<0.001

### Individual differences in respiratory response

**FIGURE 7:**
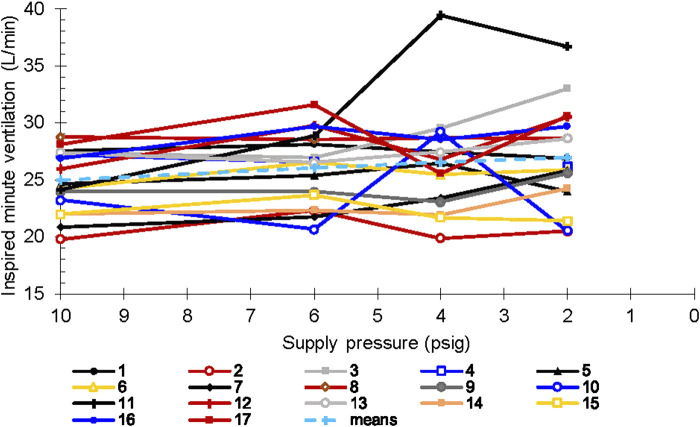
Minute ventilation *vs*. supply pressure. Each line represents a single subject.

As noted above, we did not observe changes in minute ventilation, tidal volume, or breath duration across CRU-103 supply pressures when the sample was analyzed in aggregate. However, we observed notable variation across individuals (Figure 7). Individual participants demonstrated large changes in minute ventilation, with some fractional differences greater than two coefficients of variation.

We noted similar individual differences in breath duration. Two participants increased average breath duration by more than 20% (slowed their breathing rates) for the 4 psig inlet condition only, while one increased breath duration similarly for both the 4- and 2 psig regulator inlet pressure conditions. Conversely, three participants decreased breath duration by more than 20% (breathed faster) for the 2 psig regulator inlet condition only, while one did for both the 4- and 2 psig conditions.

Work of breathing and mask pressures each demonstrated increased variance as supply pressure decreased, indicating different behaviors across participants in response to the changing supply pressures. WOB_i_/V_T_ demonstrated differences from the variance at 10 psig for all other inlet pressures: 6 psig: F(1,15) = 3.96, *p* = 0.03; 4 psig: F(1,14) = 11.4, *p* < 0.002; 2 psig: F(1,15) = 47.6, *p* < 0.0001; a marginal increase in variance from 6- to 4 psig (F(1,14) = 2.82, *p* < 0.06 and a significant increase from 4- to 2 psig CRU-103 inlet pressure (F(1,14) = 4.29, *p* < 0.03).

**FIGURE 8:**
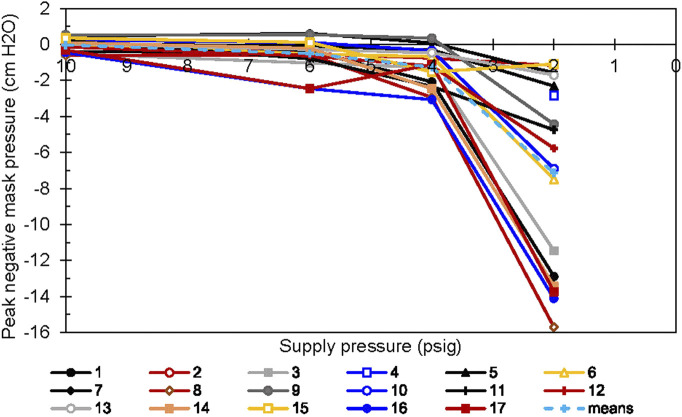
Minimum mask pressures *vs*. supply pressure. Each line represents a single subject.

We also observed significant individual differences in the minimum mask pressure during inspiration. As shown previously (Table 2), all other regulator inlet conditions had lower median minimum mask pressures than did the 10 psig condition. More striking, the spread of the values was almost 20 times greater with the 2 psig condition than with the 6 psig condition (Figure 8). For two individuals (Participants 16 and 17), the minimum mask pressure was roughly 2 cm H_2_O more negative at 6 psig than at 10 psig regulator input pressure.

**FIGURE 9:**
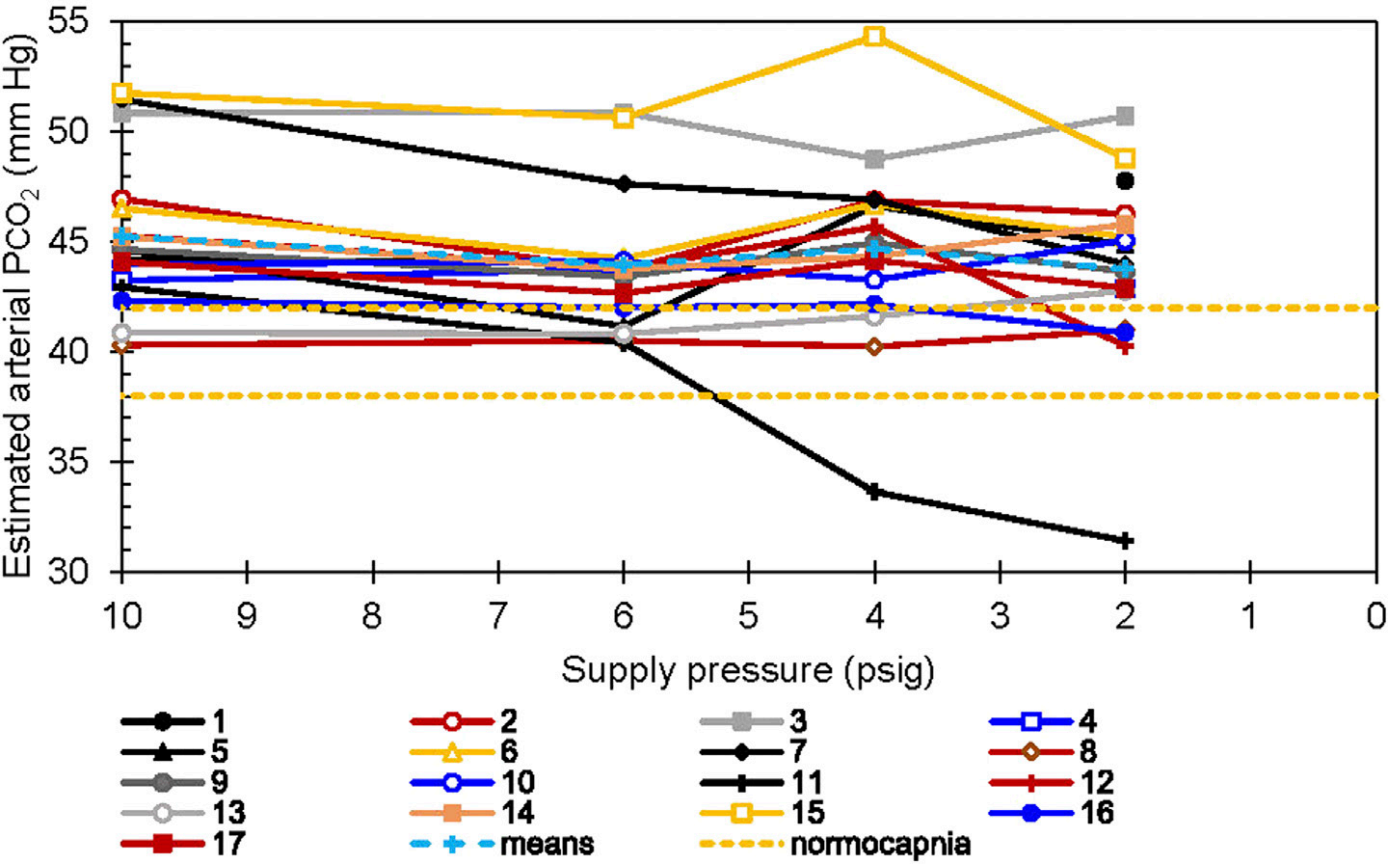
Estimated P_a_CO_2_ *vs*. supply pressure. Each line represents a single subject. The two dashed horizontal lines show the normocapnic range, 40 ± 2 mm Hg. The Jones correction (Jones et al., 1979) was applied to end-tidal measurements.

Estimated arterial carbon dioxide partial pressure (P_a_CO_2_) values are shown in Figure 9. The estimates were calculated (Jones et al., 1979) from concurrently measured end-tidal CO_2_ and tidal volume. The values did not differ significantly with regulator inlet pressure. Once again, however, individual responses varied. For Participants 3 and 15 at 6 psig and Participant 15 at 4 psig, P_a_CO_2_ was more than two standard deviations above the group means. Participant 15 had P_a_CO_2_ 7.3% higher with 4 psig than with 6 psig, but lower with 2 psig. Conversely, for Participant 11 with 2 or 4 psig at the regulator inlet, P_a_CO_2_ was more than two standard deviations below the group mean, 17 and 22% lower than that for the same individual at 6 psig regulator inlet pressure.

## Discussion

This study examined interactions among the CRU-103 regulator, the MBU-23/P mask, and the user, as well as individual differences in user respiratory response to breathing during periods of low regulator inlet pressure. Our analyses revealed two primary findings. First, the regulator and mask are independent neither of each other nor of the human breathing from the system; the respiratory dynamics of the human affect system performance and vice versa. Second, there was considerable between-subjects variability in respiratory responses to the breathing conditions studied here. We discuss these findings and their implications for tactical aviation.

### Interactions between regulator, mask, and user

We noted several systematic changes across CRU-103 inlet pressures and user respiratory behaviors. Lower CRU-103 inlet pressures were associated with elevated regulator output pressure at the end of inspiration. This increased the cracking pressure of the exhalation valve (Warkander and Arnold, 2020), adding an expiratory threshold load. Human response to expiratory valve cracking pressure may differ based on whether the system imposes expiratory threshold loading alone or adds other loads as well.

The maximum regulator outlet pressure (pressure in the mask inlet hose) occurred during expiration, yet was correlated with the absolute values of peak inspiratory flow for marginal and higher regulator inlet pressures and with minute ventilation for inadequate to acceptable regulator inlet pressures but not for higher (10 psig) inlet pressures. Because these variables occur during different respiratory phases, the correlation more likely implies an effect of breathing dynamics on regulator function than a direct relationship. The elevated regulator outlet pressure can be a consequence of degradation of internal regulator dynamics by the low pressure on the upstream side (Warkander and Arnold, 2020), leading to a delay in the cessation of flow. Further, when gas is flowing, pressure in the hose and at the mask inlet will be lower than that in the regulator. A sudden change from low to high mask pressure to start exhalation may generate overpressures in the hose, and excessive regulator outlet pressure requires a finite time to bleed down to the set point.

The variance in regulator outlet pressure during the course of the test was much greater at 2 psig than in any other condition. This finding potentially speaks to the interactions of the regulator, the mask, and the breathing patterns of the user; the user’s subjective experience of how easy or difficult it is to breathe is not entirely dependent on the supply of gas to the CRU-103, but is possibly more closely tied to supply of gas from the regulator to the mask inlet hose.

### Individual differences

Though we did not observe any statistically significant differences in minute ventilation, estimated P_a_CO_2_, and breath duration across regulator supply pressures in the sample as a whole, we found large differences in these measures across participants. Increasing variability in several measures at lower supply pressures indicates different individual responses to those conditions. The inter-individual variability in our results is an important finding. Even in a well-controlled laboratory setting, physical interactions with the LSS differed among people. When the regulator was starved for pressure in the 2 psig inlet pressure conditions, minimum mask pressure was a function of how hard the participant tried to inhale before their respiratory system adjusted its strategy. Different people can generate and/or tolerate lower inspiratory pressures than others, depending, for example, on the strength of their inspiratory muscles. They will meet their ventilatory needs with different strategies, both for that reason and also, perhaps, because of different respiratory reflex sensitivities. This may partly explain the increasing spread in minimum mask pressures as the regulator is made progressively less responsive by decreasing its inlet pressure.

The mean P_a_CO_2_ throughout this experiment corresponded to a degree of CO_2_ retention, (P_a_CO_2_ >45 Torr as compared to the normal 40 ± 2 mm Hg), by definition hypo ventilation. Some participants were hypercapnic (P_a_CO_2_ > 50 Torr). Although within-individual P_a_CO_2_ was maintained on average for all regulator supply pressures, during conditions of low supply pressure one participant retained CO_2_, and another responded by hyperventilating (Figure 7) to a moderate level of hypocapnia (P_a_CO_2__2_ < 35 mmHg) (Figure 9). However, we did not observe any correlations between respiratory patterns and reported symptoms in our data.

Hyperoxic gas affects chemical control of ventilation. Despite the mild hyperventilation sometimes reported in association with hyperoxia at rest (Becker et al., 1996; Dean et al., 2004), during exercise, hyperoxic gas may be associated with decreased $\overset{.}{V}$ relative to that with air for the same external workload (Shykoff and Warkander, 2012). Extremes of CO_2_ partial pressure values like those seen in this experiment are possible in an aircraft at similar (approximately 280 Torr) or higher inspired PO_2_. Increased inspired PO_2_ suppresses peripheral chemoreceptor output (Dahan et al., 1990), leaving the central chemoreceptors as the main or only active chemical sensors; in cats where the dose response has been well-defined, arterial PO_2_ greater than 200 Torr eliminates carotid body chemoreceptor output (Lahiri and DeLaney, 1975). With 94% oxygen from the OBOGS, the peripheral chemoreceptors can be expected to be suppressed for the entire operating range of the aircraft.

### Implications for life support systems in tactical aircraft

Modern tactical aircraft are extremely complex systems made up of many thousands of components, often designed and manufactured by separate entities. Multiple components, even if individually functioning within specified requirements, may interact with one another in unexpected ways. We believe our results reinforce the need for design specifications of individual components to accommodate as wide a range of performance environments as possible in order to account for such interactions. Our results also emphasize the importance of accounting for such interactions during evaluation testing, particularly at the extremes of possible performance conditions. Just as importantly, these results reinforce the need for acceptance standards to account for a wide range of individual variability among users, and ideally should be based on sample sizes large enough to adequately characterize people at the extremes of the population. Testing must evaluate a broad range of individuals under a broad range of respiratory conditions in order to completely understand the performance of a system.

Our findings indicate that individuals do not respond consistently to disruptions in the breathing system. Exposures that may be perfectly acceptable to one person may cause notable difficulty for another. One possible mitigation is to train pilots in certain breathing techniques to help ensure more consistent physiological status. Altering breathing patterns to accommodate exposures might help reduce some of the issues described above, and differences in breathing likely explain why some of our participants were relatively unperturbed even at the lowest regulator supply pressures. However, pilots would need to recognize potentially subtle changes in LSS output in order to adjust accordingly. The cognitive demands of tactical aviation would likely interfere with such recognition as well as with the conscious control of respiration in many cases. Further, given the individual differences we observed, it is unclear if various breathing techniques would be universally beneficial. Rather than asking pilots to adapt to the breathing system, ensuring consistency in the delivery of breathing gas is a more reliable way to help reduce any potential respiratory or physiological disruptions that may result from momentary changes to the operation of the LSS.

### Limitations and future research

One limitation to the study is our use of a baseline assessment during each test condition rather than a separate control condition. The use of a baseline measure allowed us to assess physiological changes without potential confounding factors such as day-to-day changes in hydration or circadian rhythm, and comparison across inlet pressures still allows conclusions about relative physiological effects. However, the lack of a time-matched control exposure introduces the possibility of both time- and expectancy effects, though we would not expect such factors to impact respiratory variables as easily as more subjective measures.

Although we attempted to capture as many relevant variables in this study as possible, we could not recreate the flight environment. Several additional variables that may impact physiologic response (e.g., ambient temperature, hydration status, oxygen partial pressure, barometric pressure) were not examined here. Future research should examine these additional variables and their interactions.

Finally, we do not fully understand the relevant factors associated with individual variability in our results. The link between individual traits or states and the response to various respiratory challenges will be important to investigate in future research. Understanding such interactions will help better predict and mitigate potential respiratory issues in the aircraft.

## Conclusion

This study evaluated interactions between the user, the CRU-103 regulator, and the MBU-23/P mask in response to periods of reduced supply pressure to the CRU-103. We found flow restriction and unexpected pressure overshoot from the regulator at low inlet pressures, which in turn affected user respiratory behavior and expiratory threshold loading *via* changes in mask valve operating pressures. The behavioral response to these respiratory conditions varied across participants, emphasizing the need to account for a large range of operating conditions and user behaviors during system design and testing. More research is needed to characterize the relevant factors affecting individual user behavior and effects.

## Data Availability

The raw data supporting the conclusion of this article will be made available by the authors, without undue reservation.
